# BSImp: Imputing Partially Observed Methylation Patterns for Evaluating Methylation Heterogeneity

**DOI:** 10.3389/fbinf.2022.815289

**Published:** 2022-02-10

**Authors:** Ya-Ting Sabrina Chang, Ming-Ren Yen, Pao-Yang Chen

**Affiliations:** Institute of Plant and Microbial Biology, Academia Sinica, Taipei, Taiwan

**Keywords:** bisulfite sequencing, cellular heterogeneity, imputation, methylation heterogeneity, methylation patterns, enzymatic methyl sequencing

## Abstract

DNA methylation is one of the most studied epigenetic modifications that has applications ranging from transcriptional regulation to aging, and can be assessed by bisulfite sequencing (BS-seq) or enzymatic methyl sequencing (EM-seq) at single base-pair resolution. The permutations of methylation statuses given by aligned reads reflect the methylation patterns of individual cells. These patterns at specific genomic locations are sought to be indicative of cellular heterogeneity within a cellular population, which are predictive of developments and diseases; therefore, methylation heterogeneity has potentials in early detection of these changes. Computational methods have been developed to assess methylation heterogeneity using methylation patterns formed by four consecutive CpGs, but the nature of shotgun sequencing often give partially observed patterns, which makes very limited data available for downstream analysis. While many programs are developed to impute genome-wide methylation levels, currently there is only one method developed for recovering partially observed methylation patterns; however, the program needs lots of data to train and cannot be used directly; therefore, we developed a probabilistic-based imputation method that uses information from neighbouring sites to recover partially observed methylation patterns speedily. It is demonstrated to allow for the evaluation of methylation heterogeneity at 15% more regions genome-wide with high accuracy for data with moderate depth. To make it more user-friendly we also provide a computational pipeline for genome-screening, which can be used in both evaluating methylation levels and profiling methylation patterns genomewide for all cytosine contexts, which is the first of its kind. Our method allows for accurate estimation of methylation levels and makes evaluating methylation heterogeneity available for much more data with reasonable coverage, which has important implications in using methylation heterogeneity for monitoring changes within the cellular populations that were impossible to detect for the assessment of development and diseases.

## 1 Introduction

Methylation is one of the most studied epigenetic modifications (Moore et al., 2013). It is known to be involved in a wide range of key biological processes including regulation of gene expression, developments (Hsieh et al., 2020), aging and silencing of transposable elements (Jin et al., 2011). The study of methylation at single nucleotide resolution is made possible through next generation sequencing when it is coupled with bisulfite treatment (Barros-Silva et al., 2018) or enzymatic methyl sequencing (EM-seq) (Vaisvila et al., 2021). Methylation level is used extensively in comparing between samples of different conditions (Hsieh et al., 2020) and their correlation with gene expression is usually studied (Hanley et al., 2017). When looking at reads covering multiple cytosines there are also methylation patterns, or permutations of methylation statuses spanning multiple cytosines in a row. As a read represents a cell within a bulk sequencing data, therefore the methylation patterns observed from different reads can be used to study the heterogeneity of multiple cells, i.e., cellular heterogeneity. Cellular heterogeneity was found to be closely associated with diseases. For example, in the course of cancer progression (Jin et al., 2011); the more heterogeneous the tumours are, the worse the clinical outcomes (Landau et al., 2014). A few methods had been proposed to study cellular heterogeneity. Single cell bisulfite sequencing (scBS-seq) is a typical approach; however, it is known to have significant challenges such as technical difficulty in isolating individual cells and DNA samples being destroyed by bisulfite treatment. A new method is to quantify methylation heterogeneity using methylation patterns that are formed by methylation statuses of several cytosines within the same reads in bulk sequencing data (Shannon, 1948; Hill, 1973; Zhang et al., 2011). However, owing to the nature of shotgun sequencing, the average depth and coverage in most whole genome bisulfite sequencing data (WGBS) and reduced representation bisulfite sequencing (RRBS) data are not sufficient for estimating methylation heterogeneity accurately, e.g., the depths at individual cytosine sites do not constitute enough methylation patterns at each cytosine.

Imputation is a commonly used technique to overcome this type of problems; however, most imputation methods developed for methylation data analysis are not designed for imputing the methylation patterns (haplotypes of methylation statuses). METHimpute (Taudt et al., 2018), was developed for imputing methylation levels using WGBS. Melissa (Kapourani and Sanguinetti, 2019) and DeepCpG (Angermueller et al., 2017) were developed for imputing methylation levels in single cell methylomes. Despite their usefulness in inferring methylation levels genomewide, they were not designed for and hence are unable to recover read specific methylation patterns that are needed for the estimation of methylation heterogeneity since it requires read identity for each methylation status. PReLIM (Scott et al., 2020), on the other hand, attempts to impute methylation statuses on individual sequencing reads; however, the method requires training models using many bins and the program written is not straightforward.

DNA methylation is catalysed by a family of DNA methyltransferases (DNMTs) (Jin et al., 2011). Different contexts of methylation, methylation occurring at CG, CHG, and CHH contexts where H is any of A,C, or T, are responsible by different groups of DNMTs. DNA methylation for mammalians primarily occur at CG (Jin et al., 2011) while methylation at other contexts CHG and CHH are also common for plants although their roles are not clear. Currently, most of the studies based on methylation heterogeneity are for human diseases, hence, only available for CG methylation but the same concept can be useful for other contexts as well, for example, for studying DNMTs and the pathway involved (Harris and Zemach, 2020). Therefore, it is our aim to develop an imputation method that is accurate, speedy and produces outputs widely applicable to animals as well as plants and fungi, with higher resolution (methylation pattern information).

There is high correlation of methylation among cytosines that are nearby (Affinito et al., 2020). We use this property extensively to borrow the most information from nearby sites and developed a probabilistic-based imputation method to impute accurate methylation statuses speedily. Our program is the first of its kind to be able to take any methylation contexts (not limited to CG) that has accuracy comparable with the only existing method that imputes methylation statuses. It also has the flexibility for user to specify window size in number of cytosines fixed across the genome for imputation and genomewide profiling. After all, it is easier to use, can be run with one command and outputs results readily for downstream analyses.

## 2 Methods

**FIGURE 1:**
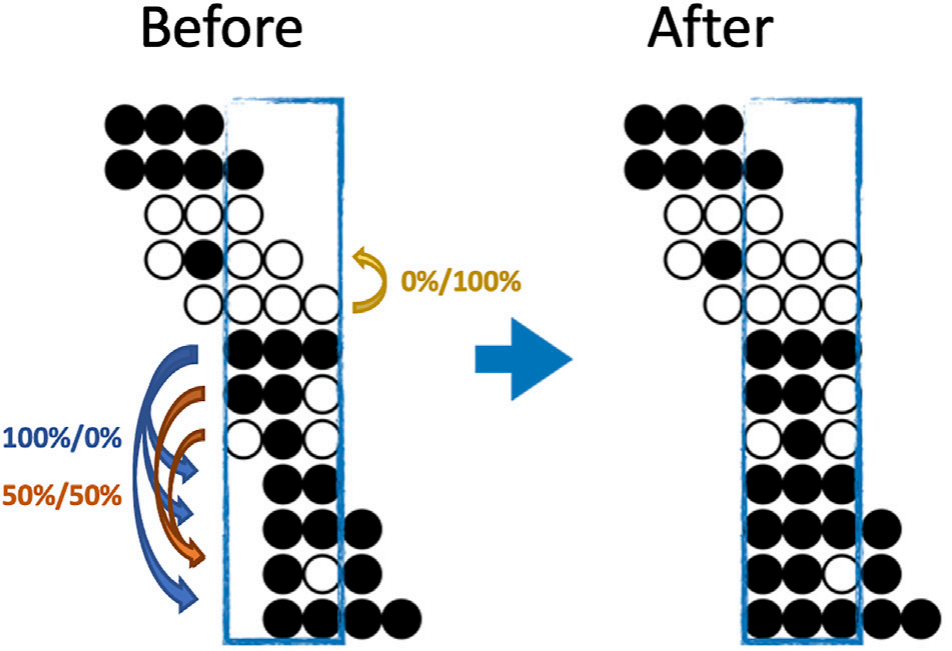
An illustration of both the eligibility and a possible imputation result for BSImp. Given we are interested in window size of three cytosines, a region is selected as enclosed by a blue rectangle. Each line of dots represents a read aligned to a specific genomic region; black (white) dots represents a methylated (unmethylated) cytosine for the given read. Four complete patterns are observed, which makes the window eligible for imputation. Within the window, only reads missing at most one methylation statuses (dots) are eligible for imputation; there are five in the example. Looking at the topmost reads with one missing pattern, the rest of the pattern resembles that of the complete pattern below, so it has a probability of zero being methylated. The second last read has methylation pattern (black, white in the second and third position) resembles two other reads, which have methylation statuses of methylated and unmethylated, one each, so it has 50% of being either.

Considering methylation patterns formed by methylation statuses of multiple successive cytosines of the same reads, there is usually missing values of methylation statuses within a window of fixed size (number of cytosines). We assume that the pattern of methylation is similar for cells within a population and that the behaviour of cells or the methylation statuses of a cell at a given position can be predicted by those statuses nearby and cells nearby; therefore, using law of total probability, let the methylation status of a cytosine at a position *j* for read *i* be *m*_*ij*_, then the probability of *m*_*ij*_ being methylated, or 1, is

$$p\left(m_{ij}=1\right)=p\left(m_{-i,j}=1|m_{i,-j}=s_{1}\right)p\left(m_{i,-j}=s_{1}\right)$$ (1)

$$\;+p\left(m_{-i,j}=1|m_{i,-j}=s_{2}\right)p\left(m_{i,-j}=s_{2}\right)$$ (2)

$$\;+\cdots +p\left(m_{-i,j}=1|m_{i,-j}=s_{n}\right)p\left(m_{i,-j}=s_{n}\right)$$ (3)

where s_*u*_ are subpatterns of complete patterns within the same window, or methylation patterns at positions other than *j*, if they exist, and *p*(*m*_*i*,*j*_ = 1|*m_i_*_,−*j*_ = s*_u_*) is the observed probability of cytosines being methylated at position *j* given subpattern *m_i_*_,−*j*_ within the window is like s_*u*_. The reads eligible for imputation is specified to be those missing at most 1 methylation status within the window. Since *m*_*ij*_ is the only missing value in the window for the same read, *m_i_*_,−*j*_ must be equal to one of s_*u*_ where *u* ⊂ {1, 2, *…* , *n*}. However, if the subpattern is not observed, or there is no complete pattern with subpattern that resembles *m_i_*_,−*j*_, it is taken as the methylation level at position *j*, or *p*(*m*_*tj*_ = 1) for all reads *t* that are observed at position *j*. An illustration of the eligibility of reads for imputation and a possible imputation result can be found in Figure 1.

In our implementation, the imputations are done alongside genome screening where windows of fixed size of cytosines of the same methylation contexts are extracted, imputed if valid and profiled for their copy numbers of methylated, unmethylated reads and every possible methylation patterns. It is done through sliding windows with *w*—1 cytosines overlapping. Only windows with at least two complete patterns are considered for imputation and results outputted if a given cytosine has enough depths or methylation levels above a threshold within the window, as specified by the user.

## 3 Result

To evaluate imputation performances, different types of data including WGBS and RRBS are used. The increased genome coverage after imputation, and the accuracy of prediction evaluated using both methylation statuses and methylation level are assessed. The WGBS data selected are from Arabidopsis thaliana and RRBS data is from human. In the evaluation only the forward strand is used.

### 3.1 Imputation can Increase Significantly in Coverage

**FIGURE 2:**
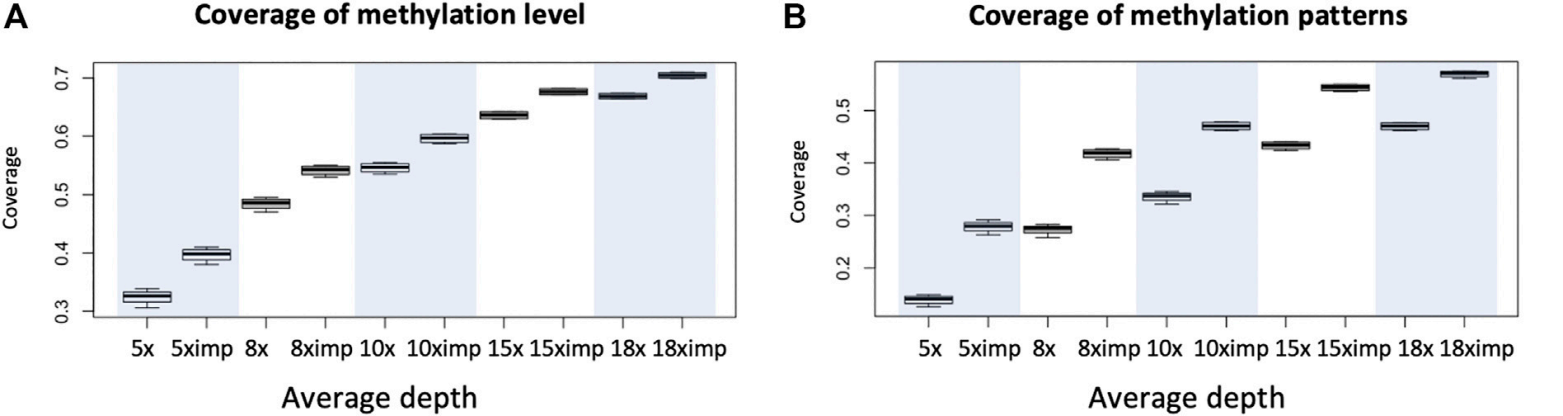
An illustration of increases in coverage for the evaluation of methylation level and methylation heterogeneity for WGBS data with common depths. **(A)** Coverage (percentage of genomic regions in terms of windows) of possible evaluation of methylation level before and after imputation evaluated with requirement of minimum depth of 8 reads at each cytosine. **(B)** Coverage (percentage of genomic regions in terms of windows) of possible evaluation of methylation heterogeneity before and after imputation using window size of 3 CpG and minimum 8 reads per window. Eight was chosen for it is the minimum number of reads required for us to see all possible patterns, if they all appear. Each boxplot is based on 1 chromosome and 4 libraries (WGBS) (4 data points). Since methylation patterns are profiled using sliding windows of one cytosine and a window is 3 CpG so coverage is calculated as the number of windows available for downstream analyses divided by total number of CpGs within the chromosome.

The primary purpose of imputation is to increase coverage genomewide for downstream analysis so we first examine the increase in coverage of our method. Four WGBS datasets with average depths of 18x are used. Data of lower depths are obtained by sampling reads based on ratios calculated as expected depths/18 to reach desirable average depths. The chosen depths are 5x, 8x, 10x, 15x and 18x. We can see a clear trend of increase in coverage as depth increases and the coverage for imputed methylomes are much higher than that before imputation with maximum linear increase above 15% for depths equals to 8. Looking at Figure 2A and Figure 2B we also see the coverage for methylation level is much higher than methylation heterogeneity; the reason for this being it is much harder to observe complete methylation patterns compared to reads at individual cytosines using the same requirements (8 reads at individual cytosine or 8 complete patterns). Also consider it usually requires only 4 reads for the evaluation of methylation levels, there is a need for imputation for the evaluation of methylation heterogeneity.

### 3.2 Imputation Predicts Methylation Statuses Accurately

**FIGURE 3:**
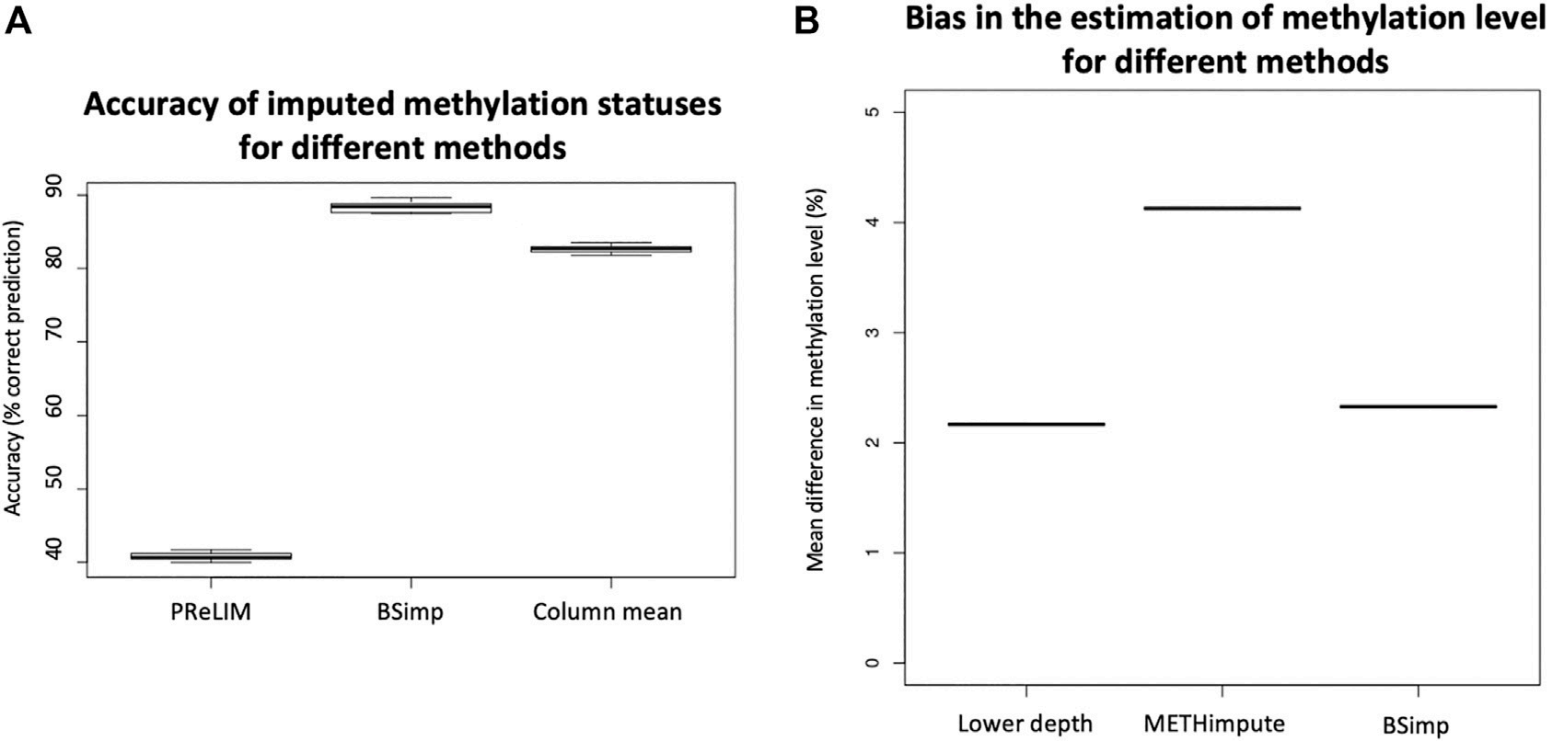
Evaluation of accuracy of BSimp by comparing with existing methods. **(A)** Boxplot of accuracy of methylation statuses imputed compared with PReLIM and column mean. Non-overlapping windows of four CGs are extracted, multiple methylation statuses removed at random and imputed using all three methods to calculate mean accuracy of prediction for each window. PReLIM needs training of models, (complete parts of) all windows included in the evaluation were used for training. Column mean is a method that uses methylation level for the genomic position as the probability of methylation for all reads eligible for imputation. The results are based on 783 windows. **(B)** Boxplot of mean difference in methylation level of common regions compared with METHimpute and data of lower depths. Four libraries were obtained by downsampling 50% of reads independently and imputed using METHimpute and BSImp. Methylation levels obtained before and after imputation are compared with library before downsampling as treating it as the correct answer. Only common regions (CG) are considered. Each boxplot is based on four results.

Given imputation increases coverage (genomic regions) for downstream analyses, it is also important to know how much bias it introduces. We first compare our method with PReLIM which is the only existing method that also recovers methylation patterns. The result as shown in Figure 3A is obtained by getting all complete patterns within windows of 4 CpGs in chromosome 2 of a human cancer data, removing multiple methylation statuses at random within each window and impute the missing values using different methods (BSImp, PReLIM and column mean) for comparison. Column mean is a method that uses methylation level for the genomic position as the probability of methylation for all reads eligible for imputation. The accuracy is calculated as the mean number of correctly imputed methylation statuses. Figure 3A shows that our method has higher accuracy (over 85%) than PReLIM and using column mean as probability for predicting methylation statuses.

Since imputation changes the estimate for methylation level at each cytosine, we also assess the accuracy (bias) using absolute changes in methylation level estimated. Figure 3B is obtained by calculating the mean absolute difference in methylation level across common regions with estimate of methylation level between original data (18x) and data of lower depth by downsampling 50% of reads and imputed data using different methods as indicated by the *x*-axis. Four libraries are obtained by downsampling 50% of the reads; each boxplot consists of result from 4 libraries. Figure 3B shows our method does not create much difference in methylation level compared to data before imputation as indicated by lower depth and METHimpute introduces much larger bias for these cytosines.

## 4 Discussion

**FIGURE 4:**
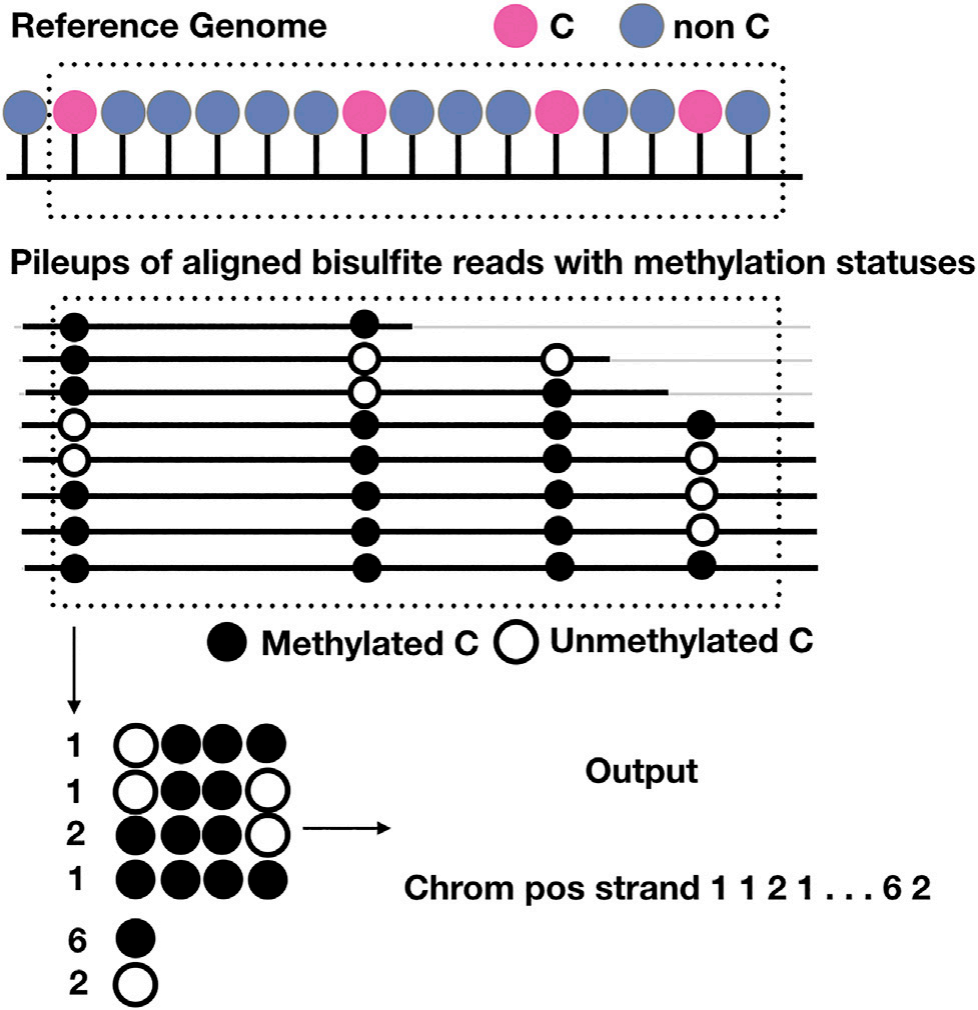
Schematic representation of the output of BSImp. First the reads are aligned to the reference genome, given any window of specified number of cytosines (here is 4), methylation statuses are extracted and missing values imputed if valid, then methylation profiled as a vector including chromosome, position, strand, the copy numbers of all possible methylation patterns starting at the position, and number of methylated and unmethylated cytosines.

There is only one existing method that recovers methylation patterns, which can be beneficial for the evaluation of methylation heterogeneity; however, the program written is standalone; it only imputes or completes a binary matrix of indicator variables that represent the methylation statuses within a window of given numbers of CpGs; it is up to the users to extract the windows for training and predicting and to output results useful for downstream analyses. On the other hand, our program (Figure 4) is able to screen for methylation pattern genomewide, impute missing statuses and output the profiles of methylation statuses at each cytosine and the copy number of every possible methylation patterns given the size of the window. In other words, it is the first of its kind and all in one. The results produced include number of methylated and unmethylation cytosine at each position given the depth is enough and the copy numbers of every possible methylation patterns starting at the same position, which can be easily used to evaluate methylation levels and methylation heterogeneity. We compared the accuracy of BSImp in terms of accurate prediction of methylation statuses with PReLIM as it is the only method that recovers methylation patterns and to our surprise, PReLIM performs a lot worse and we also ran an analysis of the breakdown of methylation levels of the windows we used in the evaluation and it turns out that PReLIM performs bad when methylation levels are high, which might requires some tuning of parameters as high methylation level can be common for most methylation contexts of interest; i.e., CpG for human.

As for methylation, existing methods that impute methylation levels were mostly developed for imputing whole methylome of sparse data such as single cell methylomes; however, METHimpute is developed for imputing methylation level of entire methylomes using WGBS, which is closer to our aim; therefore, we only compared with METHimpute using the sites with common coverage (of METHimpute and BSImp using WGBS) where BSImp has lower and the result indicates our method is comparable to METHimpute at predicting methylation level and the bias is only slightly larger than that of lower depth (data before imputation) by treating original data (not downsampled) as target.

In the evaluation we had put less emphasis on non-CpG contexts as there are not as many studies in methylation as CG; hence, fewer programs to compare with. Non-CpG methylation can be more prominent than CG for species of plants and fungi, and play important roles in their development.

Non-CpG sites are denser on the genome, meaning there are generally more non-CpG sites than CpG sites within a read, allowing for the evaluation of methylation heterogeneity using larger window sizes. This takes advantage of the complexity resulted of the possible combinations of methylation patterns. However, it would in turn requires higher depths for accurate estimation of heterogeneity. Considering there will always be reads at either ends of the windows, imputation would still be beneficial to reach desirable depths for methylation patterns.

Although the non-CpG methylation levels are generally low, we can set a parameter to place a threshold on the minimum methylation level for any given window for the consideration in our program. This speeds up the program and only outputs results in regions that might be of interest to the users. Our programs allow for systematic evaluation of methylation heterogeneity using either methylation contexts, which will make significant contributions to the understanding of their roles.

## Data Availability

Publicly available datasets were analyzed in this study. This data can be found here: NCBI Gene Expression Omnibus/GSE81407, NCBI Gene Expression Omnibus/GSE95656, and https://github.com/britishcoffee/BSImp.

## References

[B1] Affinito O. Palumbo D. Fierro A. Cuomo M. De Riso G. Monticelli A. (2020). Nucleotide Distance Influences Co-methylation between Nearby Cpg Sites. Genomics 112, 144–150. 10.1016/j.ygeno.2019.05.007 31078719

[B2] Angermueller C. Lee H. J. Reik W. Stegle O. (2017). Erratum to: DeepCpG: Accurate Prediction of Single-Cell DNA Methylation States Using Deep Learning. Genome Biol. 18, 90. 10.1186/s13059-017-1189-z10.1186/s13059-017-1233-z 28395661 PMC5387360

[B3] Barros-Silva D. Marques C. J. Henrique R. Jerónimo C. (2018). Profiling Dna Methylation Based on Next-Generation Sequencing Approaches: New Insights and Clinical Applications. Genes (Basel) 9, 429. 10.3390/genes9090429 PMC6162482 30142958

[B4] Hanley M. P. Hahn M. A. Li A. X. Wu X. Lin J. Wang J. (2017). Genome-wide DNA Methylation Profiling Reveals Cancer-Associated Changes within Early Colonic Neoplasia. Oncogene 36, 5035–5044. 10.1038/onc.2017.130 28459462 PMC5578878

[B5] Harris K. D. Zemach A. (2020). Contiguous and Stochastic Chh Methylation Patterns of Plant Drm2 and Cmt2 Revealed by Single-Read Methylome Analysis. Genome Biol. 21, 194. 10.1186/s13059-020-02099-9 32762764 PMC7412668

[B6] Hill M. O. (1973). Diversity and Evenness: A Unifying Notation and its Consequences. Ecology 54, 427. 10.2307/1934352

[B7] Hsieh J. A. Yen M. R. Chen P. Y. (2020). Epigenomic Regulation of OTU5 in Arabidopsis Thaliana. Genomics 112, 3549–3559. 10.1016/j.ygeno.2020.04.006 32298708

[B8] Jin B. Li Y. Robertson K. D. (2011). Dna Methylation: superior or Subordinate in the Epigenetic Hierarchy? Genes Cancer 2, 607–617. 10.1177/1947601910393957 21941617 PMC3174260

[B9] Kapourani C. A. Sanguinetti G. (2019). Melissa: Bayesian Clustering and Imputation of Single-Cell Methylomes. Genome Biol. 20, 61. 10.1186/s13059-019-1665-8 30898142 PMC6427844

[B10] Landau D. A. Clement K. Ziller M. J. Boyle P. Fan J. Gu H. (2014). Locally Disordered Methylation Forms the Basis of Intratumor Methylome Variation in Chronic Lymphocytic Leukemia. Cancer cell 26, 813–825. 10.1016/j.ccell.2014.10.012 25490447 PMC4302418

[B11] Moore L. D. Le T. Fan G. (2013). Dna Methylation and its Basic Function. Neuropsychopharmacology 38, 23–38. 10.1038/npp.2012.112 22781841 PMC3521964

[B12] Scott C. A. Duryea J. D. MacKay H. Baker M. S. Laritsky E. Gunasekara C. J. (2020). Identification of Cell Type-specific Methylation Signals in Bulk Whole Genome Bisulfite Sequencing Data. Genome Biol. 21, 156. 10.1186/s13059-020-02065-5 32605651 PMC7329512

[B13] Shannon C. E. (1948). A Mathematical Theory of Communication. Bell Syst. Tech. J. 27, 379–423. 10.1002/j.1538-7305.1948.tb01338.x

[B14] Taudt A. Roquis D. Vidalis A. Wardenaar R. Johannes F. Colomé-Tatché M. (2018). METHimpute: Imputation-Guided Construction of Complete Methylomes from Wgbs Data. BMC genomics 19, 444. 10.1186/s12864-018-4641-x 29879918 PMC5992726

[B16] Vaisvila R. Ponnaluri V. K. C. Sun Z. Langhorst B. W. Saleh L. Guan S. (2021). Enzymatic Methyl Sequencing Detects DNA Methylation at Single-Base Resolution from Picograms of DNA. Genome Res. 31 (7), 1280–1289. 10.1186/s12864-018-4641-x PMC8256858 34140313

[B15] Zhang Y. Liu H. Lv J. Xiao X. Zhu J. Liu X. (2011). QDMR: a Quantitative Method for Identification of Differentially Methylated Regions by Entropy. Nucleic Acids Res. 39, e58. 10.1093/nar/gkr053 21306990 PMC3089487

